# Genome and antibiotic resistance characteristics of Shigella clinical isolates in Fujian Province, Southeast China, 2005–2019

**DOI:** 10.1099/mgen.0.001325

**Published:** 2024-11-20

**Authors:** Mengying Huang, Xiaoxuan Zhang, Chaochen Luo, Haibin Xu, Yufeng Qiu, Jinsong Yang

**Affiliations:** 1 Fujian Center for Disease Control and Prevention, Fuzhou, PR China; 2 Department of Preventive Medicine, School of Public Health, Fujian Medical University, Fuzhou, PR China; 3 Fujian Provincial Key Laboratory of Zoonosis Research, Fuzhou, PR China

**Keywords:** antibiotics, multidrug resistance, phylogenetic analysis, *Shigella*, whole-genome sequencing

## Abstract

Shigellosis is a serious public health issue in many developing countries. The emergence of multidrug-resistant (MDR) *Shigella* isolates has deepened the treatment difficulty and health burden of shigellosis. China is the largest developing country in the world, but so far, the genome of MDR *Shigella* isolates has not been well characterized. In this study, 60 clinical isolates of *Shigella* spp. in Fujian Province, southeast China, from 2005 to 2019 were characterized for drug resistance phenotype, whole-genome sequencing and bioinformatics analysis. The results showed that the MDR rate of *Shigella* isolates was 100%, among which the resistance rates of cefotaxime, ciprofloxacin and azithromycin were 36.67, 21.67 and 10.00 %, respectively. The positive rate of extended-spectrum beta-lactamase (ESBL)-producing strains was 23.33%. The resistance profiles of *Shigella flexneri* and *Shigella sonnei* to some antimicrobials differed. The MDR isolates carried multiple antimicrobial resistance genes, among which *bla_CTX-M-14_* and *bla_CTX-M-15_* mediated ESBL resistance*.* ‘*ISEcp1 -bla_CTX−M_ -IS903*’ (type I) and ‘*ISEcp1 -bla_CTX−M_*’ (type II) were the most common genetic environments around the *bla_CTX-M_* genes, and plasmids containing these structures included IncFII, IncI1, IncI2 and IncN. The double gene mutation pattern of *gyr*A and *par*C resulted in a significant decrease in the sensitivity of *S. flexneri* to ciprofloxacin. The overall resistance phenotype and genotype concordance rate was 88.50%, and the sensitivity and specificity of the genotype antimicrobial susceptibility test (AST) were 93.35 and 82.53 %, respectively. However, inconsistency occurred between phenotypic and genotype profiles for a few antibiotics. Phylogenomic investigation with core genome multi-locus sequence typing (cgMLST) and SNPs were used to characterize the endemic transmission of these infections in Fujian and their evolutionary origin within the global context. For *S. flexneri*, Fujian isolates were all limited to PG3 and could be divided into three phylogenetic clusters. The ciprofloxacin-resistant strains were mainly F2a and FXv and assigned to the three clusters with different quinolone resistance-determining region mutation patterns. For *S. sonnei*, most Fujian strains belonged to Lineage III with genotype 3.7.6, except three isolates of Lineage I with genotype 1.3. The strains carrying the *bla_CTX-M_* genes were dispersed, indicating different origins of gene acquisition. Most of the circulating isolates in Fujian Province were not related to major international outbreak lineages and were only endemic to the country. In conclusion, multi-drug resistance of *Shigella* isolates in Fujian Province was serious, and genome-based laboratory surveillance will be crucial to the clinical treatment and public health measures for shigellosis.

­

**Impact Statement**

Global surveillance demonstrates that *Shigella flexneri* and *Shigella sonnei* are the predominant serogroups of *Shigella* isolates, and the increasing antimicrobial resistance in these isolates is a growing international concern, particularly the proliferation of multidrug resistance (MDR) lineages worldwide. Although China experiences a high incidence of MDR *Shigella*, systematic genome studies of these isolates remain scarce. This study compared the whole-genome sequences of MDR *Shigella* isolates from southeast China (e.g., Fujian Province) collected between 2005 and 2019. The analysis identified key differences in resistance factors between the two dominant species of *Shigella*. Additionally, the study performed a consistency analysis between genotypic and phenotypic resistance profiles, providing hitherto undocumented insights into the evolutionary trends and phylogenetic relationships of these resistant clones within this region. The results showed that the determinants of drug resistance differed between *S. flexneri* and *S. sonnei*, with a high correlation observed between drug resistance genotypes and phenotypes. Furthermore, phylogenetic analysis revealed the local persistence of ciprofloxacin-resistant *S. flexneri* and the dominance of * S. sonnei carrying bla_CTX-M_* genes with genotype 3.7.6. Our findings highlight the necessity of promoting genome-wide surveillance of *Shigella* isolates across China to provide comprehensive epidemiological, etiological, transmission evolution, and clinical data on MDR *Shigella*.

## Data Summary

The short-read sequence data have been submitted to the National Center for Biotechnology Information National Library of Medicine under BioProject accession number PRJNA1111321. The detailed genomic information of Fujian isolates and the global isolates used in this study can be found in Data S1 and S2, available in the online version of this article, respectively.

## Introduction

Shigellosis is one of the most common diarrhoeal diseases in the world, leading to an estimated 1.88 trillion infections and 1.1 million deaths annually. Seventy per cent of these deaths are related to children under 5 years old and immunocompromised patients [1,2]. *Shigella* spp. is the causative agent of shigellosis in humans. There are four species within the *Shigella* genus, which include *Shigella dysenteriae*, *Shigella boydii*, *Shigella flexneri* and *Shigella sonnei*, with the latter two being the predominant serogroups responsible for more than 90% of all bacillary dysentery cases worldwide [3]. The distribution and changes in *Shigella* composition vary by country, region and year. It was reported that *S. sonnei* affects the adult population of economically developed countries, while *S. flexneri* remains the predominant pathogenic agent in childhood diarrhoea in low- to middle-income countries. Nevertheless, with development, there is a progressive decrease in the detection of *S. flexneri* and an increase in *S. sonnei* [4,6]. Shigellosis is also an important public health problem in China; although its incidence is decreasing year by year, it is still higher than in many developed countries [7,8]. Surveillance data showed that the main isolated strains in China were *S. flexneri* and *S. sonnei*. Except for Beijing, *S. flexneri* was most prevalent in central and northern China, while *S. sonnei* was most prevalent in the east and south [7]. Fujian Province, located on the southeast coast of China with convenient transportation, is a core area of the ‘21st Century Maritime Silk Road’. With economic and social development, exchanges between Fujian and other countries are becoming closer. Although the incidence of shigellosis in Fujian is relatively low, the potential risk of *Shigella* transmission is increasing.

In the past, treatment for shigellosis relied on older antimicrobial drugs, such as tetracycline, chloramphenicol, ampicillin, trimethoprim–sulfamethoxazole and nalidixic acid [9]. However, due to selection pressure and the widespread use of mobile elements associated with resistance, multidrug-resistant (MDR) strains to these antibiotics are a common problem. In recent years, the World Health Organization recommended ciprofloxacin as the first choice for the treatment of shigellosis in adults and children, with ceftriaxone, pivmecillinam and azithromycin as the second choice [10]. Unfortunately, strains of *Shigella* resistant to antibiotics, especially ciprofloxacin, third-generation cephalosporins and azithromycin, have been found in Europe, America, Asia and many other countries [11,14]. International travel and sexual transmission among men who have sex with men (MSM) contribute to the spread of *Shigella* between countries and become a potential risk for the emergence of MDR strains [15].

Compared to traditional methods like antibiotic susceptibility testing and PCR detection of drug resistance genes, whole-genome sequencing offers several advantages for obtaining antimicrobial resistance (AMR) gene data. Whole-genome sequencing can simultaneously detect all genes and mutations, discover possible new resistance determinants, reveal resistance mechanisms and distinguish strains with the same resistance pattern caused by different mechanisms. Additionally, it provides reliable basic information on microorganisms, clear phylogenetic relationships and comprehensive information for epidemiological investigations [16,20]. In addition, genome-wide data can be stored indefinitely and analysed repeatedly at any time. Open databases such as ResFinder and the Comprehensive Antibiotic Resistance Database (CARD) also provide simple and easy-to-learn methods for predicting resistance genes [21,23].

So far, limited research exists on comparing whole-genome characteristics and drug resistance phenotypes of *Shigella* in specific regions of China, particularly regarding population structure and transmission routes. To address this gap, we aimed to contribute to the data on drug resistance and molecular epidemiology of *Shigella* in Fujian Province. We collected 60 *Shigella* strains isolated from 2005 to 2019 and performed drug resistance and phylogenetic analysis based on whole genome sequencing. The findings of this study provide a basis for effectively preventing and controlling shigellosis, conducting scientific monitoring, guiding clinical medication rationally and controlling the spread of MDR strains.

## Methods

### Study population and *Shigella* isolates

From 2005 to 2019, a total of 60 *Shigella* isolates were obtained from clinical stool samples of 60 patients (duplicate isolates from the same individual were excluded) with acute bacillary dysentery admitted to diarrhoeic clinics in four surveillance districts in Fujian Province, Southeast China. The patients’ medical records were reviewed to collect their demographic characteristics. Cases of adults with chronic diarrhoea were not included in this study. All the isolates were confirmed according to a standard protocol [24], and serological confirmation was performed by slide agglutination test with polyvalent and monovalent antisera to *Shigella* (Denka Seiken, Chuo-ku, Japan) following the manufacturer’s instructions.

### Antimicrobial susceptibility testing

The broth dilution method was used to detect the minimum inhibitory concentration (MIC) of 18 antibiotics using the Sensititre AIM system (Thermo Fisher, USA) and customized antimicrobial susceptibility test (AST) plate Gov1 and Gov2 drug susceptibility culture plates (Thermo Fisher) following the manufacturer’s instructions (http://www.trekds.com/techinfo/). The antibiotics tested and MIC ranges included ampicillin (AMP, 2–64), tetracycline (TET, 1–32), chloramphenicol (CHL, 2–64), sulfamethoxazole (SMZ, 0.25/4.75 to 8/152), cefazolin (CZO, 0.5–16), cefotaxime (CTX, 0.25–8), ceftazidime (CAZ, 0.25–8), cefoxitin (CFX, 2–64), gentamicin (GEN, 1–32), imipenem (IMI, 0.25–8), nalidixic acid (NAL, 2–64), azithromycin (AZI, 2–64), sulfisoxazole (SIZ, 32–512), ciprofloxacin (CIP, 0.03–32), levofloxacin (LEV, 0.12–8), polymyxin B (PMB, 0.5–16), cefepime (FEP, 0.25–16) and streptomycin (STR, 4–32). *Escherichia coli* ATCC 25922 was used as a quality control. The Clinical and Laboratory Standards Institute (CLSI) guideline M100-S31 was used for the determination of AMR break points and for the screening and confirmation of extended-spectrum beta-lactamases (ESBLs) [25]. Owing to the absence of a clinical break point for azithromycin in CLSI, the epidemiological cut-off values of azithromycin for *Shigella* was considered to be 16 mg l^−1^ according to the European Committee on Antimicrobial Susceptibility Testing break points [26]. MDR was defined as the emergence of resistance to three or more classes of antibiotics.

### Whole-genome sequencing and processing

Bacterial genomic DNA was extracted from pure broth cultures of *Shigella* isolates using a Bacterial Genomic DNA extraction kit (51 304, QIAGEN, Germany) according to the manufacturer’s instructions. After quality control, a 350-bp DNA fragment library was constructed using the Nextera XT v2 kit (Illumina, San Diego, USA) and sequenced for 150-bp paired-end reads on an Illumina HiSeq 2500 platform (Illumina). On average, 500 Mb of clean data was generated per strain, with a minimum sequencing depth of 150×. FastQC (v0.11.5, https://www.bioinformatics.babraham.ac.uk/projects/fastqc), SPAdes (v3.14) [27] and Prokka (v1.14.6) [28] software were used for quality control, *de novo* assembly and annotation of the sequencing data, respectively. Additionally, we representatively selected six *bla_CTX-M-14_*, *bla_CTX-M-15_* and *mph*A-harbouring isolates for long-read genome sequencing using an Oxford Nanopore MinION sequencer. Canu v1.6 [29] was used for *de novo* assemblies, and Pilon v1.24 [30] was applied to correct the sequencing errors by the Illumina sequencing data.

### Identification of resistance genes, insertion sequences and plasmids

Two free analysis websites, ResFinder (https://cge.food.dtu.dk/services/ResFinder) and CARD (https://card.mcmaster.ca/analyze/rgi), were used to predict the presence and mutations of antibiotic resistance genes and mobile genetic elements (MGEs) including plasmids. ResFinder utilizes a blast search engine with a 90% identity threshold and 60% coverage rate. The presence of insertion sequences (ISs) and plasmids was analysed using MobileElementFinder v1.0.3 (https://cge.food.dtu.dk/services/MobileElementFinder/) and PlasmidFinder v2.0.1 (https://cge.food.dtu.dk/services/PlasmidFinder/), respectively, with a discrimination threshold of 95% for both tools.

### Genotyping and population structure analysis

ShigaPass (version 1.5.0) was applied for serotype confirmation of *S. flexneri* (https://github.com/imanyass/ShigaPass) [31]. Genotyping of *S. sonnei* was performed by Sonneityping tool (version 20210201) with the hierarchical single-nucleotide variant-based genotyping scheme (https://github.com/katholt/sonneityping) and implemented in Mykrobe software version 0.9.0 [4]. The *E. coli/Shigella* EnteroBase-specific scheme cgMLST protocol was used to analyse the *Shigella* population structure. The cgMLST alleles obtained by comparison with 2513 single-copy orthologous genes were used to calculate the individual genetic distance between the genomes (https://enterobase.warwick.ac.uk), and the population structure was visualized using minimum-spanning tree [32].

### Phylogenetic analysis

#### Roary-based phylogenetic analysis

After genome annotation by Prokka v1.14.6 [28], core and accessory genes were identified and extracted. Roary v3.13.0 [33] was then used to compare the identified core genes. A maximum likelihood phylogenetic tree was generated by FastTree v2.1.10 with the General Time-Reversible (GTR) nucleotide substitution model to assess relatedness among isolate genomes, followed by visualization and annotation with ITOL v6.0 (https://itol.embl.de/).

#### SNP-based phylogenetic analysis

To understand the global phylogenetic placement of Fujian *Shigella* isolates, maximum likelihood phylogenetic trees were drawn based on 131 genomes of *S. flexneri* (local 25 isolates and 106 globally distributed isolates) and 204 genomes of * S. sonnei* (local 35 isolates and 169 globally distributed isolates), respectively, as detailed in Data S2. Briefly, a pseudo genome was constructed for each sample by substitution with the alternative allele at variant sites in the BIM Collaboration Format (BCF) file in the reference genome (*S. flexneri* 2a strain 301: GenBank entry number CP000038 and *S. sonnei* Ss046: NC_004337.2). Then, a multi-FASTA whole genome alignment of 25 *S*. *flexneri* and 35 *S*. *sonnei* samples was generated, respectively. The contig files were added to the alignment with Snippy v4.4.5 (https://github.com/tseemann/snippy), using the ‘–ctgs’ flag. Putative recombination regions were detected and masked with Gubbins v2.3.4 [34], and a maximum-likelihood phylogenetic tree was constructed using FastTree (v2.1.10) under the GTR substitution model. The tree layout was graphically edited and annotated using ITOL v6.0 (https://itol.embl.de/).

### Statistical analysis

SPSS statistical software (version 23) was used for data analysis. The chi-square test was employed to determine significant differences between variables. A *P* value of less than 0.05 was considered statistically significant.

## Results

### Demographic data of the patients

Among the patients (*n*=60, median age 33.6 years, ranged 1–83), the male–female ratio was 1.61 : 1 (37 vs. 23), 22 patients (36.67%) were younger than 5, 15 patients (25.00%) were aged 5–14, 10 patients (16.67%) were aged 15–65 and 13 (21.67 %) were older than 65. All the patients showed acute diarrhoea symptoms, followed by abdominal pain (85.0%), vomiting (68.3%), fever (61.67%), rectal tenesmus (46.7%) and nausea (28.3%). All the patients recovered after treatment.

### Serotype and genotype of the isolates

The 60 *Shigella* isolates collected during 2005–2019 were identified as 25 *S*. *flexneri* and 35 *S. sonnei* by slide agglutination test, and the identity of serotype by ShigaPass was consistent with that of slide agglutination test. In the 25 strains of * S. flexneri*, serotype 2a was the predominant serotype among the isolates (48.0%), followed by serotype X variant (Xv) (28.0%), 1a (12.0%), 2b (8%) and X (4.0%). As for genotyping of *S. sonnei*, most belonged to genotype 3.7.6 of Lineage III, except for two isolates classified as genotype 1.3 of Lineage I.

### Phenotype analysis of antibiotic resistance

All *Shigella* isolates exhibited MDR. The distribution of resistance classes showed that 8.33, 6.67, 55.00 and 30.00% of isolates were resistant to 3, 4, 5, and 6 classes of antimicrobials, respectively. Notably, all isolates were resistant to ampicillin, nalidixic acid and streptomycin but remained sensitive to polymyxin B. There were significant differences in resistance rates between the two *Shigella* species, as detailed in Table 1. *S. flexneri* had higher resistance rates to ciprofloxacin (*P*<0.001) and chloramphenicol (*P*<0.001) than *S. sonnei*, and the opposite was observed for gentamicin resistance (*P*<0.001). The rates of *S. flexneri* and *S. sonnei* being resistant to one or more first-line antibiotics (third-generation cephalosporin, ciprofloxacin and azithromycin) were 100% (25/25) and 51.43% (18/35), respectively. About 23.33% (14/60) of the *Shigella* isolates produced ESBLs, of which *S. flexneri* included Xv (*n*=3) and F1a (*n*=1), and *S. sonnei* were all genotypes 3.7.6 (*n*=10). The resistance rates of *S. sonnei* to cefotaxime and azithromycin increased significantly from 2005~ to 2015~ (from 33.33 to 100% for cefotaxime and from 3.7 to 100% for azithromycin), while the resistance rates of *S. flexneri* to ciprofloxacin and azithromycin increased more obvious from 2005~ to 2015~ (from 27.27 to 75.00% for ciprofloxacin and from 9.09 to 50.00% for azithromycin; Fig. S1).

**Table 1.** Drug resistance rates of *Shigella* isolates in Fujian Province

Antibiotic	Total (*n*=60) (%)	*S. flexneri* (*n*=25) **(%)**	*S.sonnei* (*n*=35) **(%)**	*χ* ^2^	*P* value
AMP	100	100.00	100.00	–	–
CTX	36.67	28.00	42.86	1.386	0.239
CAZ	3.33	4.00	2.86	0.059	1.000
CZO	43.33	32.00	51.43	2.242	0.134
CFX	1.67	4.00	0.00	1.424	0.417
FEP	10	12.00	8.57	0.011	0.917
IPM	3.33	4.00	2.86	0.059	1.00
NAL	100.00	100.00	100.00	–	–
CIP	23.33	52.00	2.86	19.688	<0.001
LEV	13.33	8.00	5.71	0	1.000
GEN	55.00	16.00	77.14	21.832	<0.001
CHL	35.00	84.00	0.00	45.231	<0.001
STR	100.00	100.00	100.00	–	–
TET	83.33	92.00	77.14	1.371	0.242
SMZ	76.67	68.00	82.86	1.799	0.180
SIZ	80.00	68.00	88.57	3.857	0.049
AZM	8.33	8.00	8.57	0	1.000
PMB	0.00	0.00	0.00	–	–
ESBLs	23.33	16	28.57	1.288	0.256

AMP, ampicillin; AZM, azithromycin; CAZ, ceftazidime; CFX, cefoxitin; CHL, chloramphenicol; CIP, ciprofloxacin; CTX, cefotaxime; CZO, cefazolin; FEP, cefepime; GEN, gentamicin; IPM, imipenem; LEV, levofloxacin; NAL, nalidixic acid; PMB, polymyxin B; SIZ, sulfafurazole; SMZ, sulfamethoxazole; STR, strepyomycin; TET, tetracycline

#### Determinants of resistance

Genomic analysis of the 60 *Shigella* isolates revealed a diverse array of AMR determinants. A total of 18 different resistance genes or mutations were identified across seven classes of antibiotics, including beta-lactams (*bla_TEM_*, *bla_OXA_*, *bla_CTX-M_*), quinolones (*gyr*A, *par*C, *qnr*B4), macrolides (*mph*A, *erm*B), tetracyclines (*tet*A, *tet*B, *tet*C), aminoglycosides (*aac*(*3/6*), *aadA, aph*(*3/6*)), chloramphenicol (*catA1*) and sulfamethoxazole (*sul1*, *sul2*, *dfrA;* Table S1). Additionally, 30 ISs and 16 different plasmid replicon types were found in the isolates.

### *β*-Lactam resistance genes

The major beta-lactam resistance genes identified in the 60 *Shigella* isolates were the penicillin resistance genes (*bla_OXA_*, *bla_TEM_*) and the ESBL-encoding gene (*bla_CTX-M_*). Importantly, *bla_OXA_* was mainly found in *S. flexneri* (*n*=25), all of which were *bla_OXA-1_*, and *bla_TEM_* was mainly found in *S. sonnei* (*n*=35), all of which were *bla_TEM-1_* except one *bla_TEM-128_. S. sonnei* harboured *bla_CTX-M-14_* (*n*=14), *bla_CTX-M-15_* (*n*=2) and *bla_CTX-M-64_* (*n*=1), and *S. flexneri* carried *bla_CTX-M_* included *bla_CTX-M-14_* (*n*=3) and *bla_CTX-M-55_* (*n*=1; Data S1). The proportion of *S. sonnei* carrying *bla_CTX-M_* gene (17/35) was higher than that in *S. flexneri* (4/25), *P*<0.05.

#### Quinolone resistance genes

**Fig. 1.**
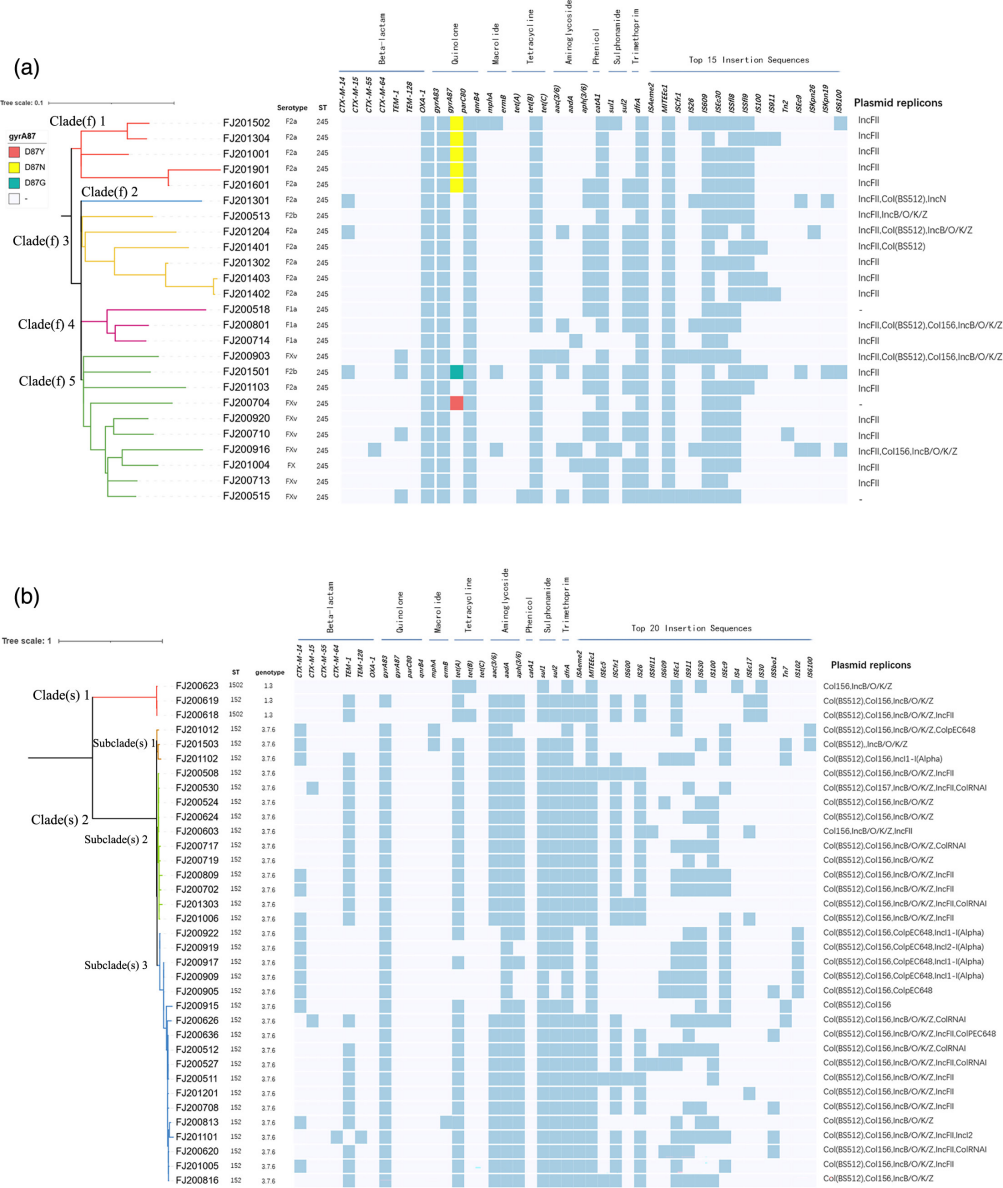
Midpoint-rooted phylogenetic tree of 25 *S. flexneri* isolates (**a**) and 35 *S. sonnei* isolates (**b**) based on the Roary analysis, and the distribution of AMR determinants. Clades(f) and Clades(s)/Subclade(s) labelled in different colours indicated the clades of *S. flexneri* and *S. sonnei*, respectively. Blue boxes represented the identity of different types of AMR genes and ISs. *GyrA* mutations, including D87Y, D87N and D87G, were separately indicated by the colours red, yellow and green. Serotype, multi-locus sequence typing (MLST) and plasmid replicons of the isolates were also listed.

Quinolone resistance-determining region (QRDR) mutations, including *gyr*A and *par*C, were found in a high proportion (e.g., >80 %) of the *Shigella* isolates. Four non-synonymous point mutations were detected in the *gyrA* gene*,* including codon *83* (*Ser to Leu*, S83L) and codon *87* (*Asp to Asn*, D87N*, Asp to Tyr,* D87Y and *Asp to Gly*, D87G). Only the Ser to Ile (S80I) point mutation was detected in *par*C gene (Data S1). Four mutation patterns, including *gyr*A (S83L) + *par*C (S80I), *gyr*A (D87N, S83L) + *par*C (S80I), *gyr*A (D87G, S83L) + *par*C (S80I) and *gyr*A (D87Y, S83L) + *par*C (S80I), were identified in *S. flexneri*, with the first being the predominant mutation pattern (*n*=18). In contrast, *S. sonnei* isolates harboured only the *gyr*A (S83L) mutation. The plasmid-mediated quinolone resistance (PMQR) gene *qnrB* was also found in *S. flexneri*, while none of the PMQR genes were detected in *S. sonnei*. The prevalence of these mutations is detailed in Fig. 1.

#### Macrolide resistance genes

Macrolide resistance genes identified in *Shigella* included *mphA* and *ermB*. The *mphA* gene displayed a broader presence, detected in both *S. flexneri* (*n*=3) and *S. sonnei* (*n*=2) isolates. In contrast, the *ermB* gene was found exclusively in a single *S. sonnei* strain (Fig. 1).

#### Tetracycline, aminoglycosides, chloramphenicol and sulfonamides resistance genes

There were significant differences in the distribution of tetracycline resistance genes *tet*(A, B) between *S. flexneri and S. sonnei* (*P*< 0.001). *S*. *flexneri* displayed a higher prevalence of *tetB* (*n*=24) compared to *tetA* (*n*=1) and *tetC* (*n*=1), whereas only *tetA* (*n*=30) and *tetB* (*n*=2) were detected in *S. sonnei* (Fig. 1)*. aac*(3/6), *aadA*(3/6) and *aph*(3/6) were predominant aminoglycoside resistance genes in *Shigella,* whose proportion in *S. sonnei* (28/35, 30/35, 34/35) was higher than that in *S. flexneri* (6/25, 15/25, 3/25), with *P* values <0.001, <0.05 and <0.001, respectively. The chloramphenicol resistance gene *catA1* was mainly found in *S. flexneri* (*n*=24). No *S. sonnei* harboured this gene. The main sulfamethoxazole resistance genes detected in *S. flexneri* were *sul2* (*n*=16) and *dfrA*, including *dfrA1* (*n*=25), *dfrA12* (*n*=2), *dfrA14* (*n*=2) and *dfrA17* (*n*=2), and the main sulfamethoxazole resistance genes detected in *S. sonnei* were *sul1* (*n*=34), *sul2* (*n*=31) and *dfrA,* including *dfrA1* (*n*=33), *dfrA12* (*n*=24) and *dfrA17* (*n*=1) (Fig. 1).

#### MGEs associated with AMR

Notably, all 60 *Shigella* strains harboured the IS MITEEc1. The top five IS elements in *S. flexneri* were MITEEc1 (*n*=25), IS609 (*n*=25), ISSf18 (*n*=22), ISEc30 (*n*=20) and ISSf19 (*n*=14). Only one isolate carried the Tn2 transposon. In contrast, *S. sonnei* displayed a wider variety of IS elements. The top eight identified were MITEEc1 (*n*=35), ISAeme2 (*n*=25), ISCfr1 (*n*=25), IS26 (*n*=22), ISEc1 (*n*=20), IS630 (*n*=19), ISEc9 (*n*=16) and IS911 (*n*=11). Four strains harboured Tn7 transposons. Except for three isolates lacking a plasmid replicon, *S. flexneri* harboured four types of plasmid replicon, with IncF (*n*=22) being the most dominant. In contrast to *S. flexneri*, *S. sonnei* displayed a higher diversity with eight identified replicon types. Notably, Col (BS512) (*n*=33), Col156 (*n*=33), IncB/O/K/Z (*n*=28) and IncF (*n*=15) were the main types found in *S. sonnei* (Fig. 1).

#### Genetic environment of *bla_CTX−M_*

Genetic environment surrounding the *bla_CTX−M_* genes can be divided into five types and named type I–V (Data S1), including type I (*n*=11) ‘*ISEcp1 -bla_CTX−M_ -IS903*’, type II (*n*=7) ‘*ISEcp1 -bla_CTX−M_*’, type III (*n*=1) ‘*ISEcp1 -bla_CTX−M_ -ORF477*’, type IV (*n*=1) ‘*ISEcp1 -bla_CTX−M_ -ΔIS903*’ and type V (*n*=1) ‘*IS26 -ISEcp1 -bla_CTX−M_ -IS903*’. The mobile element *ISEcp1* was consistently found upstream of *bla_CTX−M_ genes*, and *IS903* (complete or incomplete) was mainly included downstream of the genes. Among them, type I and type II were the predominant (38.10%, 8/21 and 33.33 %, 7/21) genetic environment of the *bla_CTX−M−14_* gene and plasmids containing these structures included IncFII, IncI1, IncI2 and IncN.

#### Consistency analysis of drug resistance phenotype and genotype

In this study, 10 commonly used antibiotics were selected to analyse the correlation between antibiotic resistance phenotypes and genotypes. A total of 600 phenotypic results were obtained from 60 *Shigella* isolates, of which 539 results exhibited phenotype–genotype consistency (overall consistency rate: 89.83%). Among the 331 resistant phenotypes, 22 lacked detectable drug-resistant genes or mutations, resulting in an overall sensitivity of 93.43%. Six isolates displayed discrepancies for β-lactams, primarily cefotaxime. The specificity of the genotypic analysis, which refers to the proportion of truly susceptible isolates lacking resistance genes, was 85.28% (Table 2). Of the 265 isolates with phenotypic sensitivity, 39 harboured drug-resistant genes or mutations. Twenty-one isolates with β-lactam resistance genes showed sensitivity to ceftazidime.

**Table 2.** Evaluation of genotype prediction for phenotypic resistance.

Antibiotics*	Phenotype: non-susceptible†(no. of isolates)		Phenotype: susceptible(no. of isolates)		Sensitivity (%）	Specificity (%）	Major resistance genes
	Genetype resistant	Genetype susceptible	Genetype resistant	Genetype susceptible			
AMP	59	1	0	0	98.33	na‡	*bla_TEM-1/128_, bla_OXA-1_*
CTX	16	6	5	33	72.73	86.84	*bla_CTX-M_*
CAZ	2	0	20	38	100	65.51	*bla_CTX-M_*
NAL	58	2	0	0	96.67	na‡	*gyr*A (S83L)
CIP	25	2	0	33	92.53	100.00	*gyr*A(S83L, D87G/D87N/ D87Y), *par*C(S80I)
AZM	6	0	0	54	100.00	100.00	*mph*A, *erm*B
SIZ	44	4	6	6	91.67	50.99	*sul*1, *sul*2
GEN	29	4	4	23	87.88	85.19	*aac*(3)-IId
TET	51	3	4	2	94.44	33.33	*tet*A/B/C
CHL	23	0	0	37	100.00	100.00	*cat*A1
Overall	313	22	39	226	93.43	85.28	

a *AMP, ampicillin; CTX, cefotaxime; CAZ, ceftazidime; NAL, nalidixic acid; CIP, ciprofloxacin (0.5 µg ml−1); AZM, azithromycin; SIZ, sulfisoxazole; GEN, gentamicin; TET, tetracycline; CHL, Cchloramphenicol.; non-susceptible, include intermediate and resistant to the antibiotics; N/A, not available.

†Non-susceptible, include intermediate and resistant to the antibiotics.

‡na, not available.

### Population structure analysis

The genetic structure of the *S. flexneri* and *S. sonnei* bacterial population was determined by the EnteroBase cgMLST scheme, and a minimum spanning tree was constructed and demonstrated, respectively (Fig. S2), which were compared with the classification results of Roary analysis. All *S. flexneri* isolates belonged to a single sequence type, ST245 (*n*=25), but the genetic distances among the five serotypes of *S. flexneri* presented distant genetic relationships (with an average of 86 different alleles). The *S. flexneri* strains classified as the same clade in Fig. 1a can be further distinguished by cgMLST analysis and exhibited distinct genetic distances, such as Clade(f) 1 (FJ201001, FJ201304, FJ201502, FJ201601 and FJ201901 of F2a) and Clade(f) 4 (FJ200518, FJ200801 and FJ200714 of F1a). The * S. sonnei* isolates were divided into two clusters according to the genotype 3.7.6 (*n*=32) and 1.3 (*n*=3), consistent with Figs 1b and S2. The *S. flexneri* and *S. sonnei* isolates with the same genotypic drug-resistant profile could be classified as the same cluster, within 20 allele differences.

### SNP-based phylogenetic analysis

**Fig. 2.**
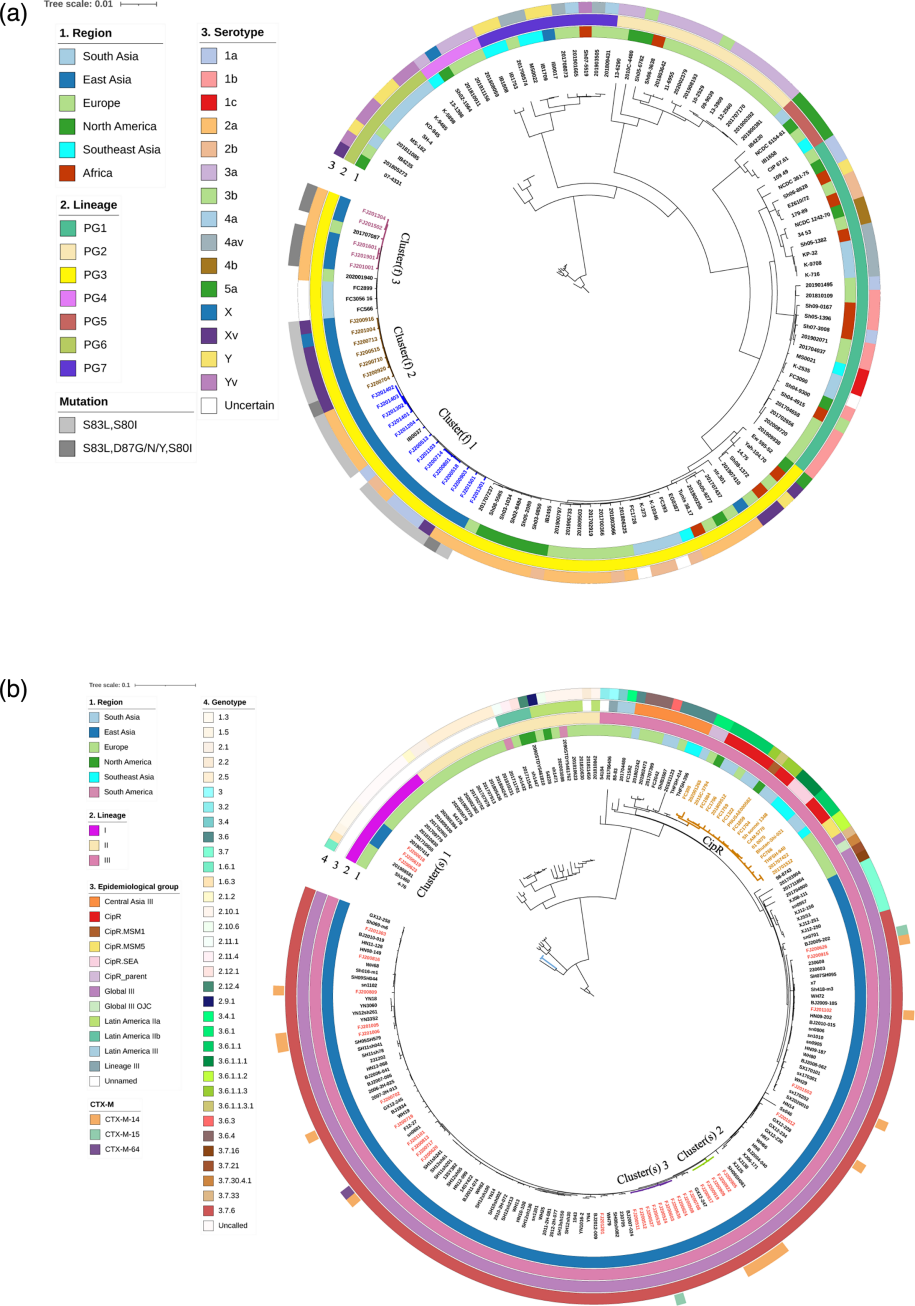
Phylogenetic tree for core-genome SNP of *S. flexneri* isolates (**a**) and *S. sonnei* isolates (**b**) in a global context. Genome 07–4331 and 4–76 were used as out-group to root the trees of *S. flexneri* and *S. sonnei*, respectively. Cluster(f) and Cluster(s) indicated the clades of *S. flexneri* and *S. sonnei*, respectively. Blue, orange and peach branches denoted Cluster(f) 1–3, while light blue, green and purple represented Cluster(s) 1–3, and gold for ciprofloxacin resistance (CipR) epidemiology groups. The annotations (**a**) were (from inner to outer ring): region of the isolates, PGs, serotype and QRDR mutation in *gyrA* and *parC*. The annotations (**b**) were (from inner to outer ring): region of the isolates, Lineage, epidemiological group, genotype and *bla_CTX-M_* gene type.

The phylogenetic tree of *S. flexneri* (Fig. 2a) showed that the 131 genomes were divided into seven distinct phylogenetic groups (PGs), and the Fujian isolates were all distributed in PG 3. The *S. flexneri* isolates in Fujian could be subdivided into three clusters, named as Cluster(f) 1, 2 and 3, and Cluster(f) 1 was the largest, containing nine isolates of 2a, three of 1a and one of Xv, exhibiting 5–191 SNPs. These isolates appeared in the 10-year period between 2005 and 2015, showing the long-term colonization characteristics of *S. flexneri* species. In addition, serotypes 2a, 1a and Xv were dispersed in Cluster(f) 1, indicating several independent serotype conversions. There were 7, 1 and 4 isolates of ciprofloxacin-resistant in Cluster(f) 1, 2 and 3, respectively, and F2a accounted for 66.67% (8/12). Nearly all isolates in the three clusters carried *gyrA* S83L and *parC* S80I mutations, but Cluster(f) 3 carried more D87N/G/Y mutations. Fujian isolates of Cluster 1 showed a close relationship to a domestic strain of China (IB0037), while Cluster 3 was clustered with an isolate from France (201707087), suggesting that these two clusters may have different evolutionary origins. Moreover, the results of phylogenetic classification based on SNP and Roary analysis were similar, for example, Cluster(f) 3 was consistent with Clade(f) 1 (including 5 F2a), while Cluster(f) 2 matched several strains in Clade(f) 5 (including 6 FXv and 1 Fx) (Figs1a  2a).

For *S. sonnei*, the 35 genomes can be ascribed to two lineages, among which Lineage I contained three strains with genotype 1.3 (named Cluster(s) 1), and Lineage III contained 32 strains with genotype 3.7.6, including two major groups (named Cluster(s) 2 and 3) (Fig. 2b), and the classification was identical to the population structure analysis (Figs 1b and S2B). Cluster(s) 2 and Cluster(s) 3 were the largest genetic branch with 7–141 SNPs, and six strains carried the *bla_CTX-M_* genes (5 of *bla_CTX-M-14_* and 1 of *bla_CTX-M-15_*). According to the classification of the epidemiological group, Cluster(s) 2 and Cluster(s) 3 belonged to Global III, which was genetically distant from the global ciprofloxacin resistance (CipR) groups, such as CipR_parent, CipR MSM5 and CipR_SEA. Except for Cluster(s) 1, the Fujian isolates and the majority of the Chinese isolates with genotype 3.7.6 were all located in Lineage III and formed a distinct Chinese subgroup.

## Discussion

The widespread use of antimicrobials has led to the emergence of MDR *Shigella*, posing a serious threat to the effective treatment of bacillary dysentery and becoming a significant burden on global public health. Currently, ciprofloxacin, azithromycin and ceftriaxone are the most commonly used antibiotics for the empirical treatment of shigellosis [1]. Worryingly, resistance to azithromycin, ciprofloxacin and third-generation cephalosporins is rising globally among *Shigella* strains [4,35, 36]. This study revealed that all *Shigella* isolates from Fujian Province were MDR, with a higher incidence compared to the reported rate in Asia (68.7%, 95 % CI: 59.9–77.5) [36]. The strains displayed 100% resistance to ampicillin, nalidixic acid and streptomycin, indicating the inadequacy of these traditional antibiotics for treating shigellosis. In our region, the resistance rates of *S. flexneri* and *S. sonnei* isolates to cefotaxime from 2005 to 2019 reached 28% (7/25) and 42.86% (15/35), respectively, which were similar to the resistance rates of *S. flexner*i (34.40%) and * S. sonne*i (43.2%) reported in Jiangsu Province of China from 2012 to 2015 [37]. The positive rate of ESBL-producing *Shigella* isolates in Fujian was 23.33% (14/60), similar to the positive rate in Jiangsu Province of China from 2013 to 2015 (25.89 %) [37]. Although this rate was higher than that reported in Africa (1.5%) [38], it was still lower than the rate observed in southwest Iran (43.0%) [39] and Barcelona of Spain (28.75%) [40]. The non-susceptible rate to ciprofloxacin of *Shigella* isolates in Fujian was 43.33% (26/60), which was comparable to the national resistance rate of 44.65% reported in China in 2014 [7]. It is noteworthy that the resistance rate of *S. flexneri* to ciprofloxacin in Fujian was 52.00%, significantly higher than that of *S. sonnei* and similar to the rate observed in Taiyuan, Shanxi Province [41]. In this study, *S. flexeri* Xv had high resistance to first-line antibiotics, among which the positive rate of ESBL-producing strains was 42.86% (3/7), the non-sensitivity rate to ciprofloxacin was 100% and the resistance rate to azithromycin was 14.29% (1/7). *S. flexneri* Xv is a newly identified serotype that is currently widespread in China, and it has also acquired resistance to a variety of antibiotics [42]. The emergence of new serotypes with enhanced resistance to first-line antibiotics may be a strategy for *S. flexneri* to maintain its epidemic capacity. The resistance rates of *Shigella* isolates in Fujian to the three first-line antibiotics showed upward trends in general, especially the rates of *S. flexneri* to ciprofloxacin and *S. sonnei* to cefotaxime, and it was similar to the global pattern [43,44]. Antibiotic usage in China is complex and widespread, and China has become the largest producer and consumer of antibiotics in the world [45]. Among them, cephalosporins, macrolides, fluoroquinolones and penicillins are the most commonly used antibiotics in China, not only for clinical treatment but also for the livestock industry [46,47]. These antibiotics may cause persistent selection pressure in the clinic, agriculture and environment, leading to the emergence and spread of severe MDR *Shigella* in China.

Historically, public health efforts to monitor and address bacterial AMR relied primarily on low-resolution phenotypic analyses. However, the affordability of whole genome sequencing is revolutionizing this approach by providing a high-resolution view of the evolution and spread of AMR [48]. Building upon drug resistance phenotypes, further understanding of the epidemiological significance of resistance-determining factors and their ability to predict drug resistance is crucial. In the present study, 18 different drug-resistant genes across seven classes of antibiotics were found in the *Shigella* isolates of Fujian. Among the β-lactam resistance genes identified, *bla_TEM_*, *bla_OXA_* and *bla_CTX-M_* were present. The genetic environment of most *bla_CTX-M-14_* included an intact *ISEcp1* 42 bp upstream of the *bla_CTX-M-14_* open reading frame and *IS903* downstream, while the genetic environment of *bla_CTX-M-15_* consisted of *ISEcp1 -bla_CTX−M_ -ORF477*, and the results were consistent with those of *S. sonnei* isolates in the Republic of Korea [49]. Fluoroquinolones, such as ciprofloxacin, are first-line antibiotics for the treatment of shigellosis. The high ciprofloxacin non-susceptible rate (MIC ≥0.5 mg l^−1^) observed in *S. flexneri* (100%, 25/25) compared to *S. sonnei* (5.71%, 2/35) can be attributed to differences in the QRDR. In *S. flexneri*, the QRDR displayed a combination pattern with dual mutations of the *gyr*A gene (one pattern was S83L, the other was D87G, D87N or D87Y) and a single mutation of the *par*C gene (S80I) [50]. In contrast, only a single mutation of the *gyr*A (S83L) gene was detected in *S. sonnei*. In China, in addition to the most common S83L mutation of the *gyr*A gene, D87N and D87G mutations have been reported in the eastern provinces [51,52]. Interestingly, the D87Y mutation was detected only in *S. flexneri* isolated from calf faeces in a northwestern province, which suggests that *Shigella* isolates of animal origin are a potential risk for the spread of fluoroquinolone resistance [53].

The consistency analysis between genotype and phenotype of *Shigella* antibiotic resistance revealed an overall coincidence rate of 88.50%. The genotypic antimicrobial susceptibility testing demonstrated sensitivity and specificity of 93.35 and 82.53 %, respectively. However, a few discrepancies between the phenotype and genotype were observed for certain antibiotics. For instance, isolates harbouring the *bla_CTX-M_* gene remained susceptible to cefotaxime and ceftazidime. The genome analysis of these isolates has reported disruption of the promoter regions of *bla_CTX-M_* due to the inversion of an IS26 element [35,40]. Additionally, variations in promoter activity due to different spacer sequences can also influence *bla_CTX-M_* expression [54].

Phylogenetic analysis allows for the comparison of local *Shigella* genome data with global population data, providing insights into the life cycle and evolutionary relationships of *Shigella* isolates. Connor’s [55] study classified *S. flexneri* into seven PGs, with PG1, PG2, PG4 and PG6 considered the oldest lineages, while PG3 and PG5 are more recent. In this study, all *S. flexneri* isolates from Fujian Province (2005–2019) belonged to PG3 lineages, forming locally colonized populations with genetic diversity. The ability of independent serotype conversions, such as serotype 2a, 1a and Xv dispersed in cluster(f) 1, might contribute to the long-term success of *S. flexneri* locally [55]. Fujian cluster(f) 3 was clustered with an isolate from France (201707087). These findings indicated that the epidemic of *S. flexneri* in Fujian was caused by both local expansion and interregional transmission. For *S. sonnei*, five distinct lineages have been identified globally, with Lineage III emerging as the dominant lineage worldwide over the past two decades [4]. Clades 3.6 and 3.7 are the most common (19 and 62 %, respectively) and account for the majority of *S. sonnei* in all continents except Latin America [4]. In our study, the majority of *S. sonnei* isolates in Fujian Province belonged to Lineage III with genotype 3.7.6 and clustered with the majority of the Chinese isolates, forming a distinct Chinese clade (main Chinese clade) [56]. The phylogenetic tree revealed that Fujian isolates were genetically distant from the recent emergence of MDR clones of central Asian sub-lineage III (with genotypes 3.6.0, 3.6.2, 3.6.3 and 3.6.4) and CipR (with genotype 3.6.1.1 and its sub-genotypes), which are considered as part of a global pandemic [12]. These differences in the genetic distance might explain the low resistance to ciprofloxacin of Fujian *S. sonnei* at gene level. The Fujian isolates carrying multiple types of drug resistance genes (such as *bla_CTX-M_* gene) were widely distributed in the China clade, suggesting that the MDR *S. sonnei* existed in co-circulation domestically. Notably, *bla_CTX-M-14_* and *bla_CTX-M-15_* were the dominant *bla_CTX-M_* types identified in this study, contrasting with the increasing prevalence of *bla_CTX-M-27_* observed in developed countries [57,59]. The heterogeneous geographical distribution of *bla_CTX-M_* may be attributed to the differences in circulating clones between regions.

This study has several limitations that should be acknowledged. First, the selection of 60 representative *Shigella* isolates from Fujian Province may have limited the captured diversity of genome features. Moreover, due to self-medication practices in mild bacillary dysentery cases, it was not possible to obtain and analyse the genome characteristics of *Shigella* isolates associated with diarrhoea in this population. Second, due to the lack of detailed epidemiological background data and the difficulty of conducting retrospective investigations, substantial information about cases (such as travel information) might be subject to omissions and mistakes, so it could not provide epidemiological evidence for the determination of international imports.

In conclusion, this study provided insights into the antibiotic resistance of *Shigella* and the AMR determinants they carried in Fujian Province, Southeast China. The results showed that the prevalence of MDR *Shigella* was significant in Fujian Province, and the resistance rates to first-line antibiotics were on the rise. There was high consistency between AMR determinants and antibiotic resistance phenotypes. The antibiotic resistance of *Shigella* to cefotaxime, azithromycin and ciprofloxacin may be driven by a bla*_CTX-M-_* and mphA(ermB)-encoding plasmid and double/triple mutations in the QRDR, respectively. It also revealed a high degree of genetic diversity in the circulating *Shigella* strains, and there may be possible interaction between *S. flexneri* and international strains, while *S. sonnei* was dominated by local circulating clones, belong to China-specific clade. Continuous surveillance and further genomic epidemiological studies with diverse sources of *Shigella* strains are urgently needed to provide the basis for transmission risk assessment, clinical treatment and vaccine strategies.

## supplementary material

10.1099/mgen.0.001325 Uncited Table S1.

10.1099/mgen.0.001325 Uncited Fig. S1.

10.1099/mgen.0.001325 Uncited Supplementary Data Sheet 1.

10.1099/mgen.0.001325 Uncited Supplementary Data Sheet 2.

## Abbreviations

AMR: antimicrobial resistance
AST: antimicrobial susceptibility test
CARD: Comprehensive Antibiotic Resistance Database
cgMLST: core genome multi-locus sequence typing
CLSI: the Clinical and Laboratory Standards Institute
ESBLs: extended-spectrum beta-lactamases
GTR: General Time-Reversible
IS: insertion sequences
MDR: multidrug-resistant
MGE: mobile genetic elements
MIC: minimum inhibitory concentration
MSM: men who have sex with men
PMQR: plasmid-mediated quinolone resistance
QRDR: quinolone resistance-determining region
