# Supplementary material for: Genome and antibiotic resistance characteristics of Shigella clinical isolates in Fujian Province, Southeast China, 2005–2019
Source: Microb Genom. 2024 Nov 20;10(11):001325. doi: 10.1099/mgen.0.001325 (PMC11893363; doi:10.1099/mgen.0.001325)
Supplement: Uncited Table S1. [file mgen-10-01325-s001.pdf]

Supplementary table 1 The AMR genotypes and phenotypes of 60 isolates of *Shigella*

No. of AMR Phenotypes	*S. flexneri*		*S. sonnei*	
	**Phenotypic pattern(N)**	**AMR genes**	**Phenotypic pattern(N)**	**AMR genes**
**3**	0		SIZ/AMP/NAL（1）	sul2/tetA/AAC(3)-Ⅱd/blaTEM-1/gyrA（1）
			CTX/AMP/NAL（4）	blaCTX-M-14/gyrA（1）
				blaCTX-M-14/sul2/tetA/gyrA（3）
**4**	CTX/AMP/CIP/NAL（1）	gyrA/parC（1）	SIZ/TET/AMP/NAL（1）	blaCTX-M-14/sul2/tetA/AAC(3)-Ⅱd/blaTEM-1/gyrA（1）
			SIZ/GEN/AMP/NAL（1）	sul2/tetA/AAC(3)-Ⅱd/blaTEM-1/gyrA（1）
**5**	SIZ/TET/AMP/CHL/NAL（6）	sul2/tetB/OXA-1/catA1/gyrA/parC（4）	SIZ/TET/GEN/AMP/NAL（16）	sul2/tetA/AAC(3)-Ⅱd/TEM-1/gyrA（6）
		sul2/tetB/TEM-1/OXA-1/catA1/gyrA/parC（1）		sul2/tetA/AAC(3)-Ⅱd//gyrA（2）
		CTX-M-14/sul2/tetB/ACC(6')-Ⅰb10/OXA-1/catA1/gyrA/parC（1）		CTX-M-15/sul2/tetA/AAC(3)-Ⅱd//gyrA（1）
	TET/AMP/CHL/CIP/NAL（4）	tetB/OXA-1/catA1/gyrA/parC（3）		sul2/tetA、tetB/AAC(3)-Ⅱd/TEM-1（1）
		sul2/tetB/OXA-1/catA1/gyrA/parC（1）		sul2/tetA/AAC(3)-Ⅱd、ACC(6')-Ⅰb10/TEM-1（1）
				sul2/tetA/ACC(6')-Ⅰb10/TEM-1/gyrA(1）
				tetB（1）
				sul2/tetB/AAC(3)-Ⅱd/TEM-1/gyrA（2）
				sul2/tetA/AAC(3)-Ⅱd/TEM-1（1）
			SIZ/TET/CTX/AMP/NAL（2）	CTX-M-14/gyrA（1）
				CTX-M-14/sul2/gyrA（1）
				CTX-M-14/sul2/tetA/AAC(3)-Ⅱd/TEM-1/gyrA（1）

**6**	SIZ/TET/CTX/AMP/CHL/NAL（1）	tetB/OXA-1/catA1/gyrA/parC	SIZ/TET/GEN/AMP/CIP/NAL（1）	sul2/tetA/AAC(3)-Ⅱd/TEM-1/gyrA
	SIZ/TET/GEN/AMP/CHL/NAL（2）	sul2/tetA、tetB/AAC(3)-Ⅱd/TEM-1/catA1/gyrA/parC	SIZ/TET/CTX/GEN/AMP/NAL（4）	CTX-M-14/sul2/AAC(3)-Ⅱd/TEM-1/gyrA
	SIZ/TET/CTX/GEN/AMP/NAL（2）	tetB/OXA-1/catA1/gyrA/parC		CTX-M-14/sul2/tetA/AAC(3)-Ⅱd/TEM-1/gyrA
		sul2/tetB/OXA-1/catA1/gyrA/parC	SIZ/CTX/GEN/AMP/NAL/AMZ（1）	CTX-M-14/sul1/AAC(3)-Ⅱd/gyrA/mphA
	TET/CTX/AMP/CHL/CIP/NAL（1）	tetB、tetC/TEM-1/OXA-1/catA1/gyrA/parC		
	SIZ/TET/AMP/CHL/CIP/NAL（4）	sul2/tetB/OXA-1/catA1/gyrA/parC		
		CTX-M-14/sul2/tetB/OXA-1/catA1/gyrA/parC		
	TET/AMP/CHL/CIP/NAL/AZM（2）	CTX-M-14/sul2/tetB/AAC(3)-Ⅱd、ACC(6')-Ⅰb10/TEM-1/OXA-1/catA1/gyrA/parC/mphA		
		sul1/tetB/OXA-1/catA1/gyrA/parC/mphA		
**7**	SIZ/TET/CTX/GEN/AMP/NAL/AZM（1）	CTX-M-55/sul1/tetB/AAC(3)-Ⅱd/OXA-1/catA1/gyrA/parC/mphA	SIZ/TET/CTX/GEN/AMP/NAL/AZM（2）	CTX-M-14/sul2/tetA/AAC(3)-Ⅱd/TEM-1/OXA-1/gyrA/ermB
				CTX-M-14/sul2/tetA/AAC(3)-Ⅱd/mphA
	CAZ/SIZ/TET/CTX/GEN/AMP/NAL（1）	sul2/tetB/OXA-1/catA1/parC/mphA	CAZ/SIZ/TET/CTX/GEN/AMP/NAL（1）	CTX-M-64/sul2/tetA/tetA/AAC(3)-Ⅱd/TEM-128/gyrA

Note: AMP, ampicillin; CTX, cefotaxime; CAZ, ceftazidime; NAL, nalidixic acid; CIP, ciprofloxacin; AZM, azithromycin; SMZ, sulfamethoxazole; GEN, gentamicin; TET, tetracycline; CHL, Chloramphenicol.
