# Supplementary material for: Genome and antibiotic resistance characteristics of Shigella clinical isolates in Fujian Province, Southeast China, 2005–2019
Source: Microb Genom. 2024 Nov 20;10(11):001325. doi: 10.1099/mgen.0.001325 (PMC11893363; doi:10.1099/mgen.0.001325)
Supplement: Uncited Fig. S1. [file mgen-10-01325-s002.pdf]

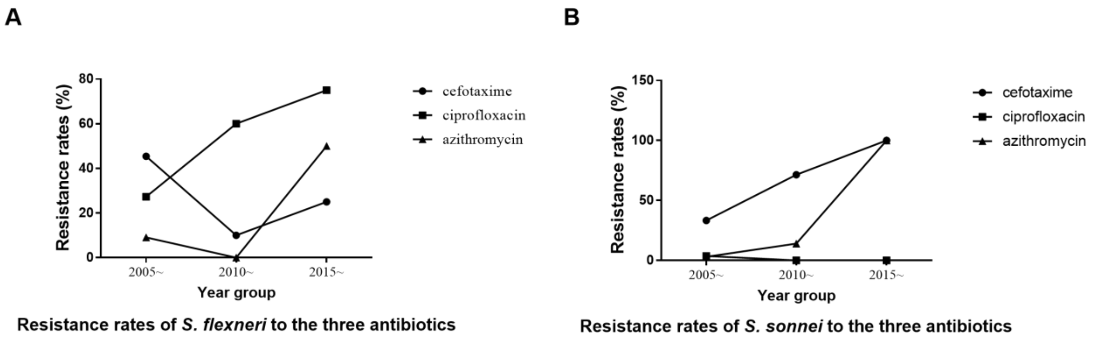

Supplementary figure 1

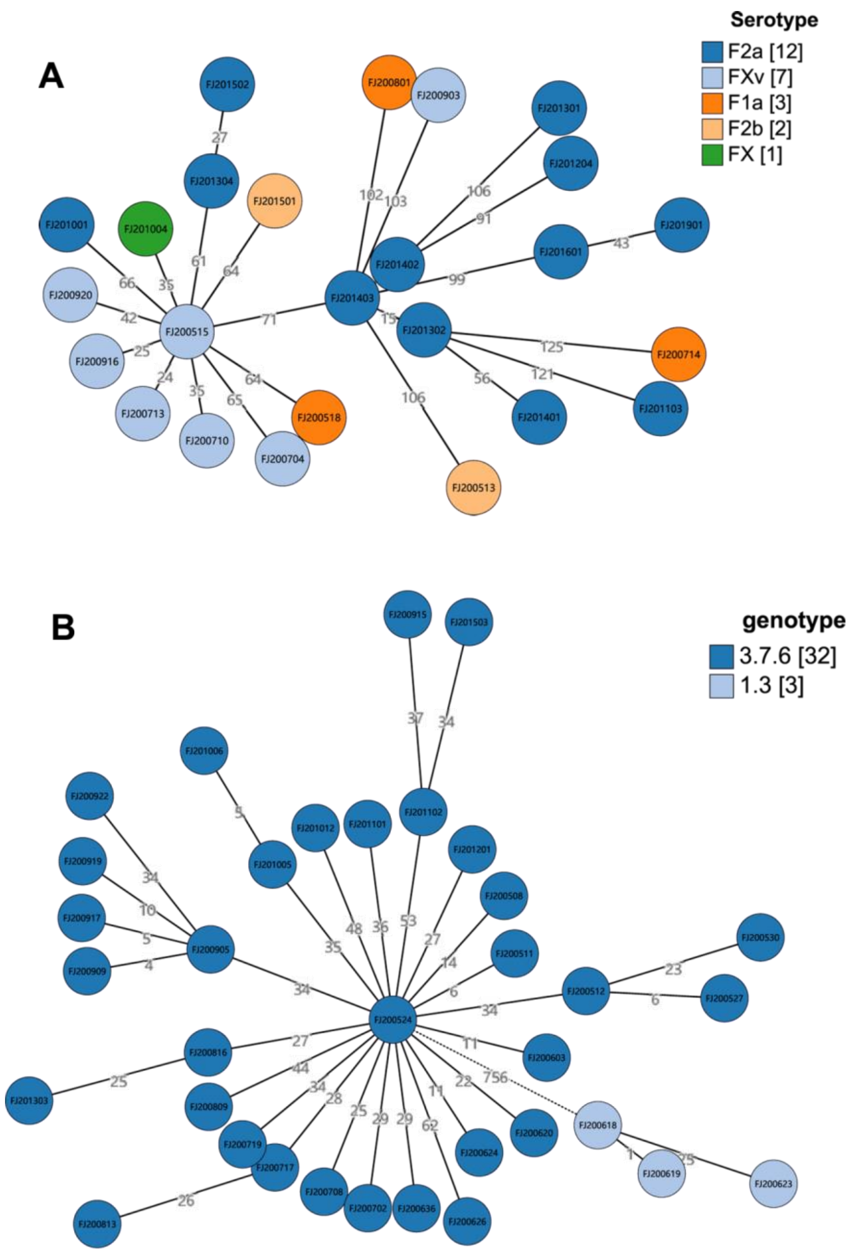

Supplementary figure 2

Supplementary Figure 2. Minimum spanning trees constructed according to the core genome allele profiles of the 25 *S. flexneri* (A) and 35 *S. sonnei* (B). The nodes were color-labeled according to the

isolates' serotype of *S. flexneri* or genotype of *S. sonnei*. The label inside the node indicated the strain code. The genetic distance between different isolates was labeled on the branches, and branches with a length greater than 50 were shortened.
